# Seroprevalence of Chikungunya Virus after Its Emergence in Brazil

**DOI:** 10.3201/eid2404.171370

**Published:** 2018-04

**Authors:** Juarez P. Dias, Maria da Conceição N. Costa, Gubio Soares Campos, Enny S. Paixão, Marcio S. Natividade, Florisneide R. Barreto, Martha Suely C. Itaparica, Cristina Goes, Francisca L.S. Oliveira, Eloisa B. Santana, Neusa S.J. Silva, Carlos A.A. Brito, Laura C. Rodrigues, Silvia Inez Sardi, Ramon C. Saavedra, Maria Glória Teixeira

**Affiliations:** Instituto de Saúde Coletiva, Rua Basílio da Gama, Salvador, Brazil (J.P. Dias, M.C.N. Costa, E.S. Paixão, M.S. Natividade, F.R. Barreto, M.S.C. Itaparica, M.G. Teixeira);; Instituto de Ciências da Saúde, Av. Reitor Miguel Calmon, Salvador (G.S. Campos, S.I. Sardi);; London School of Hygiene and Tropical Medicine, London, UK (E.S. Paixão, L.C. Rodrigues);; Secretaria Municipal da Salvador, Salvador (M.S.C. Itaparica);; Faculdade de Tecnologia e Ciências,Av. Artêmia Pires Freitas, Sim, Brazil (C. Goes);; Secretaria Municipal de Saúde de Feira de Santana, Feira de Santana, Brazil (F.L.S. Oliveira, E.B. Santana, N.S.J. Silva);; Secretaria de Saúde do Estado da Bahia, Salvador (R.C. Saavedra);; Universidade Federal de Pernambuco, Pernambuco, Brazil (C.A.A. Brito)

**Keywords:** chikungunya virus, CHIKV, survey, seroprevalence, incidence, attack rate, mosquito, viruses, vector-borne infections, Brazil

## Abstract

Chikungunya has had a substantial impact on public health because of the magnitude of its epidemics and its highly debilitating symptoms. We estimated the seroprevalence, proportion of symptomatic cases, and proportion of chronic form of disease after introduction of chikungunya virus (CHIKV) in 2 cities in Brazil. We conducted the population-based study through household interviews and serologic surveys during October–December 2015. In Feira de Santana, we conducted a serologic survey of 385 persons; 57.1% were CHIKV-positive. Among them, 32.7% reported symptoms, and 68.1% contracted chronic chikungunya disease. A similar survey in Riachão do Jacuípe included 446 persons; 45.7% were CHIKV-positive, 41.2% reported symptoms, and 75.0% contracted the chronic form. Our data confirm intense CHIKV transmission during the continuing epidemic. Chronic pain developed in a high proportion of patients. We recommend training health professionals in management of chronic pain, which will improve the quality of life of chikungunya-affected persons.

Chikungunya is an emerging mosquitoborne disease caused by an alphavirus (chikungunya virus; CHIKV) from the *Togaviridae* family (*1*). Reports of outbreaks of chikungunya were not frequent until the beginning of the 21st century; however, some serologic studies provided evidence that CHIKV had circulated in a sylvatic cycle in nonhuman primates (*2*). In 2004, an outbreak of chikungunya emerged in some Indian Ocean islands (Comoros, Seychelles, and Mauritius) (*2*) and later spread to Reunion Island; the 266,000 recorded cases represented an attack rate of 35% (*3*). In the following years, the disease circulated in Italy, France, and India (*3*), and in 2013 it was introduced in the Americas, causing an epidemic of nearly 2.6 million autochthonous cases in >40 countries from the southern United States to Argentina through December 2017 (*4*). Along with Zika and dengue viruses, chikungunya virus has become a substantial global public health threat, not only because of the high magnitude of the epidemics but also because it can produce highly debilitating clinical symptoms, including intense joint pain that can last for years (*2*). 

Brazil confirmed autochthonous cases of chikungunya in 2014 simultaneously in 2 regions: Northern, in Oiapoque/Amazonas, caused by Asian genotype virus; and Northeast, in 2 cities (Feira de Santana and Riachão do Jacuípe-Bahia), caused by the East/Central/South African (ECSA) genotype (*5**,**6*). In 2015, there were 20,661 notified cases of this disease; 271,824 cases were notified in 2016 and 171,930 cases in 2017 through epidemiologic week 35. The disease has affected >50% (2,829/5,570) of Brazilian municipalities (*7*).

In the Americas, scientific investigation on CHIKV seroprevalence is scarce; to date, only 6 studies have been published. Three of the published studies involved blood donors; 1 in Puerto Rico, with seroprevalence of 23.5% (*8*); 1 in Guadeloupe, with 48.1% seroprevalence; and 1 in Martinique, with 41.9% seroprevalence (*9*). Another study used a convenience sample of a single laboratory of Saint Martin, whose seroprevalence was 16.9% (*10*), and the fifth used a sample from a small rural area population in Brazil, where seroprevalence was 20% (*11*). The only study in South America involving random sampling of urban population was in Nicaragua, where the seroprevalence was 32.8% (*12*). Thus, we consider it important to produce information on seroprevalence of CHIKV in urban areas of Brazil.

We estimated the seroprevalence of CHIKV 1 year after the introduction of the virus in the population of 2 urban areas in Brazil, Feira de Santana and Riachão do Jacuípe-Bahia, that are affected by the ECSA genotype. We further report the proportion of symptomatic cases of chikungunya and the proportion of patients that had the chronic form of disease.

## Methods

### Study Design and Participants

**Figure 1:**
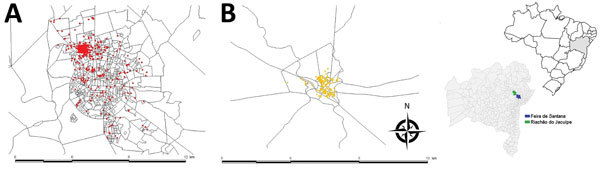
Notified cases of chikungunya georeferenced by household in Feira de Santana (A; n = 1,339), and Riachão do Jacuípe (B; n= 1,536), Bahia state, Brazil, during epidemiologic week 32 of 2014 through week 11 of 2015. Inset maps show locations of Feira de Santana and Riachão do Jacuípe in Bahia state and Bahia state in Brazil.

We conducted a cross-sectional population-based study through household interviews and serologic survey during November–December 2015, involving residents in delimited areas of 2 cities, Feira de Santana and Riachão do Jacuípe. These cities, which reported early epidemics of chikungunya in Brazil caused by ECSA genotype (*5**,**6*), are situated in Bahia state in northeastern Brazil (Figure 1), which has a hot, semiarid climate. In 2014, Feira de Santana had 612,000 inhabitants and a population density of 457.4 inhabitants/km^2^; Riachão do Jacuípe had 35,322 inhabitants and a population density of 28.5 inhabitants/km^2^ (*13*). These cities did not have *Aedes albopictus* mosquito infestations; the only vector present was *Ae. aegypti*. The mean of Premise Index (PI) of *Ae. aegypti* mosquitoes in 2014 was 1.1% in Feira de Santana and 2.0% in Riachão do Jacuípe, according to the records of the Vectors Control Program of the Health Department of Bahia State in Salvador, Bahia.

**Figure 2:**
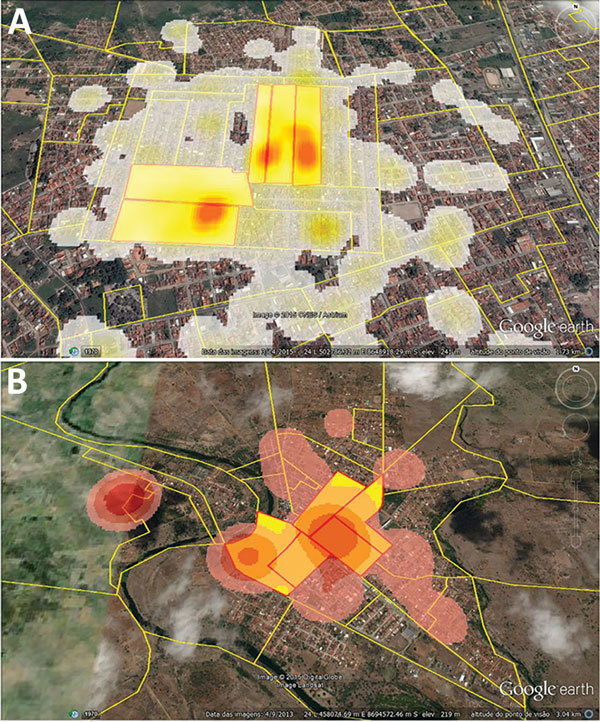
Density of notified cases of chikungunya by census tract in A) Feira de Santana and B) Riachão do Jacuípe, Bahia state, Brazil, during epidemiologic week 32 of 2014 through week 11 of 2015. Yellow shaded areas with red borders indicate the census tracts in which most cases were concentrated.

The study sampling frame was the general population of the 2 cities living in the epicenter area of the early chikungunya epidemics. To identify these epicenters, we georeferenced the cases of chikungunya notified to the Department of Health of the 2 municipalities, from epidemiologic week (EW) 32 of 2014 to EW 11 of 2015, using the geographic network of the cartographic base of each city. We then plotted the cases in the respective census tracts and estimated the kernel density (*14*) to delimit the census tracts that reported the highest number of cases (Figure 2).

Fieldworkers visited all households in the selected areas (areas with the highest density of cases, or hotspots). For the general population survey, we invited all household members who were >1 year of age to take part in the study and have a questionnaire answered if they had lived in the city for the last >6 months. We estimated the sample size for this survey as 296 persons in the delimited area of Feira de Santana and 295 in the delimited area in Riachão do Jacuípe, and estimated 30% seroprevalence, 95% confidence, and 80% power. The Research Ethics Committee of the Institute of Collective Health/Federal University of Bahia approved this study (no. 986.229 of 03/03/2015).

### Procedures

For the general population survey, we conducted interviews using a semistructured questionnaire installed on tablets to register demographics, socioeconomics, household characteristics, and health status, especially self-reported chikungunya infection. We trained undergraduate students of health to visit all households in the area, explain the project objectives, and, when allowed, collect participants’ signatures in the Free and Informed Consent Form (FICF) and carry out the interviews. For participants <18 years of age, we used the Term of Assent and a responsible adult signed the document.

After interviewing all residents in each house, we randomly selected 1 to participate in the serologic survey. This random sample was without replacement. Thereafter, a laboratory technician used a specific FICF and collected 5 mL of blood per venipuncture, in accordance with current biosafety standards, from consenting participants. Local laboratory staff separated the serum by centrifugation and conditioned it at −20°C. We transported the aliquots to the virology laboratory of the Federal University of Bahia/Institute of Health Sciences, where we processed these samples. We used ELISA (Euroimmun, Lübeck, Schleswig-Holstein, Germany) to identify specific antibodies against CHIKV, IgM, and IgG, according to the manufacturer’s instructions (*15*). We considered any person with CHIKV, IgM, or IgG (or both) detected in the serum as CHIKV infected. The Euroimmun chikungunya IgM kit considers a ratio between the extinction value of the sample by the extinction value of the calibrator given within the kit. The lower detection limit of the CHIKV ELISA is ratio 0.05 IgM and 0.06 IgG. Samples with ratio <0.8 are considered negative, and those >1.1 are positive.

To calculate the proportion of symptomatic persons, we considered those who declared they have had chikungunya disease; those who reported fever and arthralgia as disease symptoms, at minimum, from August 2014 through the interview date; and those who had IgM- or IgG-positive tests. If the symptoms of joint pain, especially in extremities, had persisted for >3 months after the onset of the disease, we considered them in the chronic phase (*16*). We considered asymptomatic those who reported they had not had CHIKV infection but who had positive results for CHIKV IgM, IgG, or both.

### Statistical Analysis

We analyzed the data using SPSS version 22 (https://www.ibm.com/analytics/data-science/predictive-analytics/spss-statistical-software). We reported the incidence of CHIKV infection (total and according to the variables of interest) as percentages with a 95% CI. We used the Pearson *χ*^2^ test to assess statistically significant differences (p<0.05) for categorical variables.

## Results

**Figure 3:**
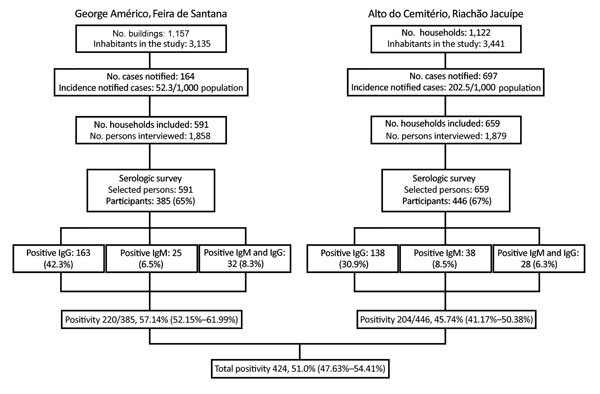
Flowchart of serologic survey of chikungunya in residents of George Américo, Feira de Santana, and Alto do Cemitério, Riachão de Jacuípe, in Bahia state, Brazil, 2015. Number ranges in paretheses indicate 95% CIs.

We georeferenced, by household, 1,339 cases of chikungunya in Feira de Santana and 1,536 in Riachão do Jacuípe (Figure 1) and identified the 5 census tracts with the highest density of cases of this disease during the epidemic in each city (Figure 2). The Municipal Department of Health of Feira de Santana registered 164 suspected cases of chikungunya disease in the selected 5 census tracts in George Américo neighborhood, corresponding to incidence of 52.3 cases/1,000 inhabitants. In Riachão do Jacuípe, the Department of Health reported 697 suspected cases in Alto do Cemitério neighborhood, corresponding to incidence of 202.5 cases/1,000 inhabitants (Figure 3).

In the selected 5 census tracts of George Américo, there were 1,157 buildings (including uninhabited households and commercial spaces) and 3,135 inhabitants in a surface area of 0.283 km^2^ (11,081.7 inhabitants/ km^2^). During the survey, we visited 591 households and interviewed 1,858 persons. In the selected 5 census tracts of Alto do Cemitério, there were 1,122 buildings in total and 3,441 inhabitants in a surface area of 0.606 km^2^ (1,454.9 inhabitants/km^2^). In this area, we visited 659 households and interviewed 1,879 persons (Figure 3).

In George Américo, we invited 591 persons to be part of the serologic survey; 385 (65%) consented. We identified 220 (57.1%) persons, 80 male (36.4%) and 140 female (63.6%), who had CHIKV antibodies: 163 IgG, 25 IgM, and 32 both. In Alto do Cemitério, the consent rate was similar, 446/659 persons (67.7%). We identified 204 (45.7%) persons, 59 (28.9%) male and 145 (71%) female, who had CHIKV antibodies: 138 IgG, 38 IgM and 28 both. We considered 9 participants in each city (18 total) as negative for CHIKV because their CHIKV ELISA ratio was 0.8–1.1 (Table; Figure 3). The difference in the seroprevalence rates of the 2 study areas was statistically significant (p = 0.001). The differences in distribution of seroprevalence by age group and sex between the study areas were not statistically significant.

**Table:** Seroprevalence of CHIKV among survey participants reporting symptomatic, asymptomatic, and chronic infection. Bahia state, Brazil, 2015–2016*

Patient characteristics	George Américo, Feira de Santana				Alto do Cemitério, Riachão do Jacuípe		
No. participants	No. positive	Prevalence, %	No. participants	No. positive	Prevalence, %
Age group, y†							
1–19	88	46	52.3		45	17	37.8
20–39	123	72	58.5		109	55	50.5
40–59	121	69	57.0		144	63	43.8
>60	53	33	62.3		148	69	46.6
Total	385	220	57.1		446	204	45.7
Sex							
M	129	80	62.0		127	59	46.5
F	256	140	54.7		319	145	45.5
Total	385	220	57.1		446	204	45.7
Serologic results							
IgG	385	163	42.3		446	138	30.9
IgM	385	25	6.5		446	38	8.5
IgM and IgG	385	32	8.3		446	28	6.3
Total	385	220	57.1		446	204	45.7
Self-reported chikungunya infection							
Yes‡	220	72	32.7		204	84	41.2
No§	220	148	67.3		204	120	58.8
Total	220	220	100.0		204	204	100.0
Self-reported chronic form¶							
Yes	72	49	68.1		84	63	75.0
No	72	23	31.9		84	21	25.0
Total	72	72	100.0		84	84	100.0

*Selected area of each city. The difference in the seroprevalence rate of the 2 areas was statistically significant (p = 0.001). CHIKV, chikungunya virus. †Children <1 y of age were not included in the study. ‡Attack rate of symptomatic CHIKV infection. §Attack rate of asymptomatic CHIKV infection. ¶Percentage among positive symptomatic cases of CHIKV infection.

Of the 220 persons with positive serologic test results in George Américo, 72 (32.7%) reported having been affected by chikungunya (Table). The rate of symptomatic CHIKV infections was significantly higher in women (54/140, 38.6%) than in men (18/80, 22.5%) (p = 0.015). For specific age groups, the difference was statistically significant (p<0.001); participants 40–59 years of age (32/69, 46.4%) and >60 years age (19/33, 57.6%) had the highest prevalence among those participants who reported symptoms. In Alto do Cemitério, the rate of symptomatic cases was 41.2% (84/204 participants) (Table). The prevalence of symptomatic cases in women (61/145, 42.1%) was also higher than in men (23/59, 39.0%), but the difference was not statistically significant (p = 0.755). For specific age groups, similar to George Américo, the difference was statistically significant (p<0.034); the 40–59-year (28/63, 44.4%) and >60-year (35/69, 50.7%) age groups had the highest prevalence among those participants who reported symptoms.

Among the cases of symptomatic CHIKV infection, the proportion of participants with symptoms that lasted >3 months (chronic chikungunya) was 49/72 (68.1%) in George Américo and 63/84 (75.0%) in Alto do Cemitério (Table). Considering the total number of study participants with positive serologic test results for CHIKV, the proportion of infected persons with long-term manifestations of the disease was 49/220 (22.2%) in George Américo and 63/204 (30.9%) in Alto do Cemitério. These proportions were 49/385 (12.7%) in George Américo and 63/446 (14.1%) in Alto do Cemitério when calculated for the whole study population in each area.

The rate of chronic forms of chikungunya disease among symptomatic participants with positive CHIKV serologic test results was higher in women than in men in both George Américo (40/54, 74.1% in women; 9/18, 50.0% in men) and Alto do Cemitério (54/61, 88.5% in women; 9/23, 39.1% in men). This difference was statistically different in Alto do Cemitério (p<0.001), whereas the p value was borderline in George Américo (p = 0.058). Regarding the age of the symptomatic persons with positive CHIKV serologic test results, we found no statistically significant difference in the rate of chronic form in both study areas.

## Discussion

Our population-based seroprevalence study of CHIKV conducted in urban areas of 2 cities in Brazil where the ECSA (*5**,**6*) genotype recently emerged showed that >45% of the study population had CHIKV antibodies. The results of CHIKV serosurveys previously conducted in countries on different continents have varied from 10.2% to 75% seroprevalence (*17**,**18*), depending on the type of population studied and the time and intensity of the virus circulation. Our study revealed a higher incidence of this virus infection, more than double the values reported in studies previously published in the Americas (*8**–**11*), except for the serosurveys conducted in Guadeloupe and Martinique, which showed values similar to ours; however, only adults eligible for blood donation were included in the population of that study (*9*). Our study indicates that all age groups and genders were similarly exposed to CHIKV infection in both George Américo and Alto do Cemitério, as supported by the absence of significant difference in CHIKV seroprevalence across those groups.

We conducted our survey 1 year after the introduction of CHIKV in this area; the high incidence of infection suggests that the transmission of CHIKV was fast and intense. In addition, CHIKV circulation seems to have remained active, because CHIKV IgM was detected in >6% of the participants. However, some studies have shown that, unlike other arboviruses, CHIKV IgM may persist for prolonged periods: 12–13 months (*19*) or up to 3 years (*20*) postinfection. Moreover, CHIKV RNA was found in perivascular synovial macrophages in 1 patient 18 months after infection, suggesting that the virus may persist in organs or sites of immune privilege (*21*). Thus, late detection of CHIKV IgM may represent prolonged viral circulation or the persistence of these antibodies in persons infected several months ago. 

We cannot extrapolate our results for the entire population living in these 2 cities because the surveys took place in areas with the highest density of chikungunya cases during the first months of the epidemic, possibly constituting the diffusion poles of CHIKV. However, over time, other areas were becoming secondary sources of transmission, which resulted in the second epidemic wave, as other researchers have observed in Feira de Santana (*22*). It is possible that the mean seroprevalence of CHIKV in the populations of Feira de Santana and Riachão do Jacuípe has reached levels closer to those we found in the areas where most chikungunya cases were reported at the beginning of the epidemic. This same pattern of spread was observed after the emergence of dengue in urban spaces (*23*)*,* evidencing once more the high vectorial capacity of the *Ae. aegypti* mosquitoes responsible for these epidemics (*5*).

The difference observed in the levels of seroprevalence between the 2 study areas may be due to different levels of vector infestation, human population density, or both. Data from the Brazilian Institute of Geography and Statistics show that the population density in the hotspots of Feira de Santana was 7 times higher than in the study area of Riachão do Jacuípe (*13*). However, the Premise Index recorded for *Ae. aegypti* mosquitoes in 2015 was lower in Feira de Santana than in Riachão do Jacuípe. 

The wide range in CHIKV seroprevalence worldwide could be explained by many reasons, such as climatic factors, vector control measures applied before and during outbreaks that affect levels of *Ae. aegypti* infestation, the previous level of immunity of the population, and the strain and genotype of CHIKV. The CHIKV seroprevalence data in our study reflect levels that may be found in other big cities, because it was a population-based survey conducted in urban areas. The 3 surveys in the Caribbean (in Martinique, Guadeloupe, and Puerto Rico) used a convenience sample or were conducted in a rural community; low population density is not the most favorable environment for the proliferation of *Ae. aegypti* mosquitoes, which are adapted mostly to urban areas (*24*).

The proportion of symptomatic persons among those CHIKV positive in our study was similar to the proportion reported by Sissoko et al. (*25*) in Mayotte (37.2%). Our study revealed that the rate of symptomatic CHIKV infections was higher in the 40–59- and >60-year age groups, similar to the results of other studies. However, despite the higher rate of symptomatic CHIKV infections in women than in men, this difference was significant only in George Américo. It is possible this difference reflects the levels of prior prevalence of rheumatologic diseases in women in the 2 study populations.

The prevalence of long-term manifestations of CHIKV varies widely, ranging from 3% among pediatric patients <15 years of age (*26*) to 83% in a cohort of rheumatology patients (*27*). A recently published metaanalysis estimated that ≈50% of chikungunya patients have long-lasting related symptoms (*28*). In our study, the proportion of participants who had long-term manifestations of the disease among persons with positive serologic test results and who reported symptoms of chikungunya reached 68.1% in George Américo and 75.0% in Alto do Cemitério. Our results are similar to those obtained in a cohort of rheumatology patients in Reunion Island (France) (*27*), in which, 1 and 2 years after disease onset, ≈80% of patients had rheumatic manifestations associated with chikungunya. Our results also indicate that, 1 year after the beginning of the CHIKV outbreak, >12% of persons in our study populations in both cities suffered from joint pain that precluded the performance of many habitual activities, affected their quality of life, required medical care, and increased absenteeism at work. Some studies have shown that the chronic phase of chikungunya can last for >2 years (*27**–**30*). Thus, because of extremely debilitating symptoms during the acute phase of chikungunya disease, elevated attack rates, and the high proportion of patients who develop long-lasting symptoms, CHIKV outbreaks can have a substantial impact on the overall health of the population.

Our serologic survey has some limitations. First, we conducted it in the epicenters of the epidemic, so the results could be overestimated and cannot be extrapolated to the whole city. The fact that symptoms were self-reported is a potential source of bias, because participants may not accurately remember the symptoms of a disease that occurred 1 year earlier. In addition, we did not collect indicators of vector infestation in the 2 study areas. Despite these limitations, our investigation is a relevant study to understand the transmission of CHIKV of the ECSA genotype after its introduction into an urban population, in which the seroprevalence corresponds to the rate of infection attack.

This study revealed a worrying scenario that emerged after the introduction of CHIKV in Feira de Santana and Riachão do Jacuípe. The virus has spread almost completely through the cities (*22**,**31**,**32*), and at least one third of the infected persons had symptoms. In addition, there was a high frequency of the chronic form of chikungunya. These results reveal how much the emergence of CHIKV in these 2 cities has altered the health status of a relevant portion of the population, especially women. It is possible that this is the scenario in many urban centers in Brazil that have experienced epidemics of CHIKV infection. Studies have shown that several factors are strongly associated with risk of chronic chikungunya, including female sex, age >45 years, acute disease severity, and presence of anterior articular disease (*19**,**27**,**33**,**34*). Despite the evidence, there is still no physiopathogenic explanation. However, because rheumatic diseases are more prevalent in women (*35*), it is plausible that CHIKV is a potential trigger for the onset or cornification of arthralgia in a group more susceptible to rheumatic diseases. Intense and disabling articular pain is a common symptom of CHIKV infection; this pain does not always respond to routine medical drugs and so requires specific and often long-term pharmacologic treatment. In chikungunya epidemic situations, the problems for patients do not end after the acute phase and decline of the transmission; a large proportion of those affected require continuous treatment and adequate pain management with drugs that can cause serious side effects, such as corticosteroids, opiates, and chemotherapeutics (*36**,**37*), to minimize suffering. These sequelae also influence individual and collective productive capacity and affect the public health sector and the economy. It is urgent to expand national and international research for developing technologies and strategies for increasing the effectiveness of vector control programs, for synthesizing antiviral drugs to mitigate the symptoms and chronic evolution of the disease, and, especially, for producing vaccines that can be used in populations vulnerable to CHIKV.
